# Population structure in diverse pepper (*Capsicum* spp.) accessions

**DOI:** 10.1186/s13104-023-06293-3

**Published:** 2023-02-25

**Authors:** Jack McCoy, Natalia Martínez-Ainsworth, Vivian Bernau, Hannah Scheppler, Grant Hedblom, Achuyt Adhikari, Anna McCormick, Michael Kantar, Leah McHale, Lev Jardón-Barbolla, Kristin L. Mercer, David Baumler

**Affiliations:** 1 Department of Horticulture and Crop Science, The Ohio State University, Columbus, OH USA; 2 Centro de Investigaciones Interdisciplinarias en Ciencias Y Humanidades, Universidad Nacional Autónoma de México, Mexico City, Mexico; 3 North Central Region Plant Introduction Station, Agriculture Research Service, United States, Department of Agriculture and Department of Agronomy, Iowa State University, Ames, IA USA; 4 Department of Food Science and Nutrition, University of Minnesota-Twin Cities, St. Paul, MN USA; 5 Department of Tropical Plant & Soil Sciences, University of Hawaii at Manoa, Honolulu, HI USA

**Keywords:** Species complex, Admixture, Sweet, Pungent

## Abstract

**Background:**

Peppers, bell and chile, are a culturally and economically important worldwide. Domesticated *Capsicum spp*. are distributed globally and represent a complex of valuable genetic resources.

**Objectives:**

Explore population structure and diversity in a collection of 467 peppers representing eight species, spanning the spectrum from highly domesticated to wild using 22,916 SNP markers distributed across the twelve chromosomes of pepper.

**Results:**

These species contained varied levels of genetic diversity, which also varied across chromosomes; the species also differ in the size of genetic bottlenecks they have experienced. We found that levels of diversity negatively correlate to levels of domestication, with the more diverse being the least domesticated.

**Supplementary Information:**

The online version contains supplementary material available at 10.1186/s13104-023-06293-3.

## Objective

Domestication and globalization have significantly affected crop diversity. Domesticated plants have long been of interest to those exploring natural selection [1]. Crop populations are subject to the same evolutionary forces that impact genetic diversity in the wild: gene flow, drift, selection, mutation, and assortative mating [2]. However, in cultivated populations, human management can influence these factors, resulting in a combination of natural and human-mediated evolutionary change. Landraces, domesticates that have not undergone improvement by modern plant breeding methods, present ideal populations in which to explore diversification and genetic structure [3]. Traditional landrace varieties often retain a higher genetic diversity than elite lines [4]. The degree of domestication can also impact genetic diversity, and this is difficult to measure precisely as it is often a continuous rather than discrete process. For instance, in chile peppers (*Capsicum* spp. L.), four levels of domestication are typically identified: wild, semi-wild, landrace, and commercial peppers [5]. Nevertheless, while genome wide levels of diversity are higher amongst landraces than commercial peppers, there can be stronger (e.g., Chile de Agua) or weaker (e.g., Mirasol) fixation for all the classic domestication syndrome traits within specific lineages of landraces and the degree to which they hybridize with wild peppers also varies [6].

Chile pepper (*Capsicum* spp.) is a culturally and economically valuable vegetable, spice, and medicine worldwide [7]. Peppers are one of the most popular vegetables across the world, and they are consumed raw, cooked, and dried for use as a spice by nearly 25% of the world’s population [8, 9]. Peppers are known to have very rich vitamin content, as well as producing a notable heat [10, 11]. There are five domesticated species in genus *Capsicum* that form a species complex which make up the crop we call pepper [12]. These species are *Capsicum annuum*, *C. chinense*, *C. frutescens*, *C. baccatum*, and *C. pubescens*. The chile pepper species complex is cultivated worldwide [12], with *C. annuum* and *C. chinense* considered to have been subject to the greatest degree of selection and demographic pressure [13]. The spread of pepper around the world has expanded its traditional uses, leading to new phenotypes and population genetic structure relative to landraces in the center of origin [14]. The present study sampled named chile peppers collected from all over the world representing eight different *Capsicum* species—the five domesticates plus *C. chacoense, C. eximium,* and *C. praetermissum—*to assess a broad range of cultivated and wild genetic resources. These peppers sample a wide range of phenotypes, uses, and cultivation histories. Thus, the objective of this study was to explore the population structure and genetic diversity of 467 chile pepper accessions from diverse genetic backgrounds.

## Data description

### Plant material, culture, and phenotypic evaluation

Pepper accessions were sourced from various seed producers across North America (Additional file 2: Table S1). In total, this study included 467 accessions spanning eight species [*C. annuum* (n = 294), *C. baccatum* (n = 33), *C. chacoense* (n = 2), *C. chinense* (n = 119), *C. eximium* (n = 3), *C. frutescens* (n = 12), *C. praetermissum* (n = 1), *C. pubescens* (n = 3)]. Two replicate plants of each accession were grown in in an RCBD in greenhouses at the University of Minnesota in summer 2018 in five-gallon containers using a standard potting mix under fluorescent lights followed by metal halide lighting. For each accession, healthy young leaf tissue was harvested from the healthiest plant for DNA extraction. The soil was fertilized with Pure Blend Pro Grow (3-2-4), Pure Blend Pro Bloom (2-3-5), and Cal-Mag Plus (all from Botanicare, AZ, USA) for a ten-week period following the manufacturer’s recommendations.

### Sequencing and SNP calling

DNA was extracted from the 467 accessions grown at the University of Minnesota representing eight *Capsicum* species using Qiagen DNeasy kit (Qiagen Ltd, Germantown, MD, USA) following the manufacturer’s instructions. All samples were sent to the University of Minnesota Genomics Center where libraries for double-digest genotyping-by-sequencing (GBS) [15] were constructed using Apek1 and Btg1 restriction enzymes. The libraries were sequenced using Illumina Novaseq 6000. Fastq files were demultiplexed using Illumina bcl2fastq software. Trimmomatic was used to remove the first 12-bases (adapter sequences) from the beginning of each read [16]. Cleaned reads were aligned to the *C. annuum* reference genome (UCD-10X-F1; a cross between Criollos de Morelos 334 landrace and a non-pungent blocky pepper-breeding line; [17]) using BWA-mem [18]. Variants were called using Freebayes software to jointly call variants across all samples [19]. The initial VCF file was filtered using VCFtools to remove variants with minor allele frequency  < 1%, variants with genotype rates  < 95%, and samples with genotype rates  < 10%. This generated a total of 22,916 SNPs across the 12 chromosomes (Additional file 1: Fig. S1). All raw sequence data can be found in the NCBI SRA under the project name PRJNA876725 (Table 1).

**Table 1:** Data associated with this manuscript

Label	Name of data file	File type	Data repository and identifier
Data set 1	pepper collection	.fastq	NCBI Sequence Read Archive: https://identifiers.org/bioproject:PRJDB3 [20]
Data set 2	pep_12_chrom.vcf	.vcf	Zenodo: https://doi.org/10.5281/zenodo.7268809 [21]
Data set 3	Filtered10percentminorallele_2966markers_77genotypes.hmp	.hmp	Zenodo: https://doi.org/10.5281/zenodo.7268809 [21]
Data set 4	Vitamin_phenotype.txt	.txt	Zenodo: https://doi.org/10.5281/zenodo.7268809 [21]

### Population genetics

Principal component analysis (PCA) was conducted with R package SNPRelate using 22,916 markers to visualize the overall distribution of genetic diversity and discern population structure related to species [22]. In addition, a neighbor joining tree was constructed using the r-package fastreeR. Diversity was explored in four common cultivated species (*C. annuum* (n = 294), *C. baccatum* (n = 33), *C. chinense* (n = 119), *C. frutescens* (n = 12)) by calculating nucleotide diversity (π) with TASSEL [23] using a sliding window of 100 markers sliding by every individual marker, but not for the four rarer species. Differences between species π related to patterns of demography (i.e., species) were determined by conducting a Dunn multiple comparison test.

### Population structure

**Fig. 1:**
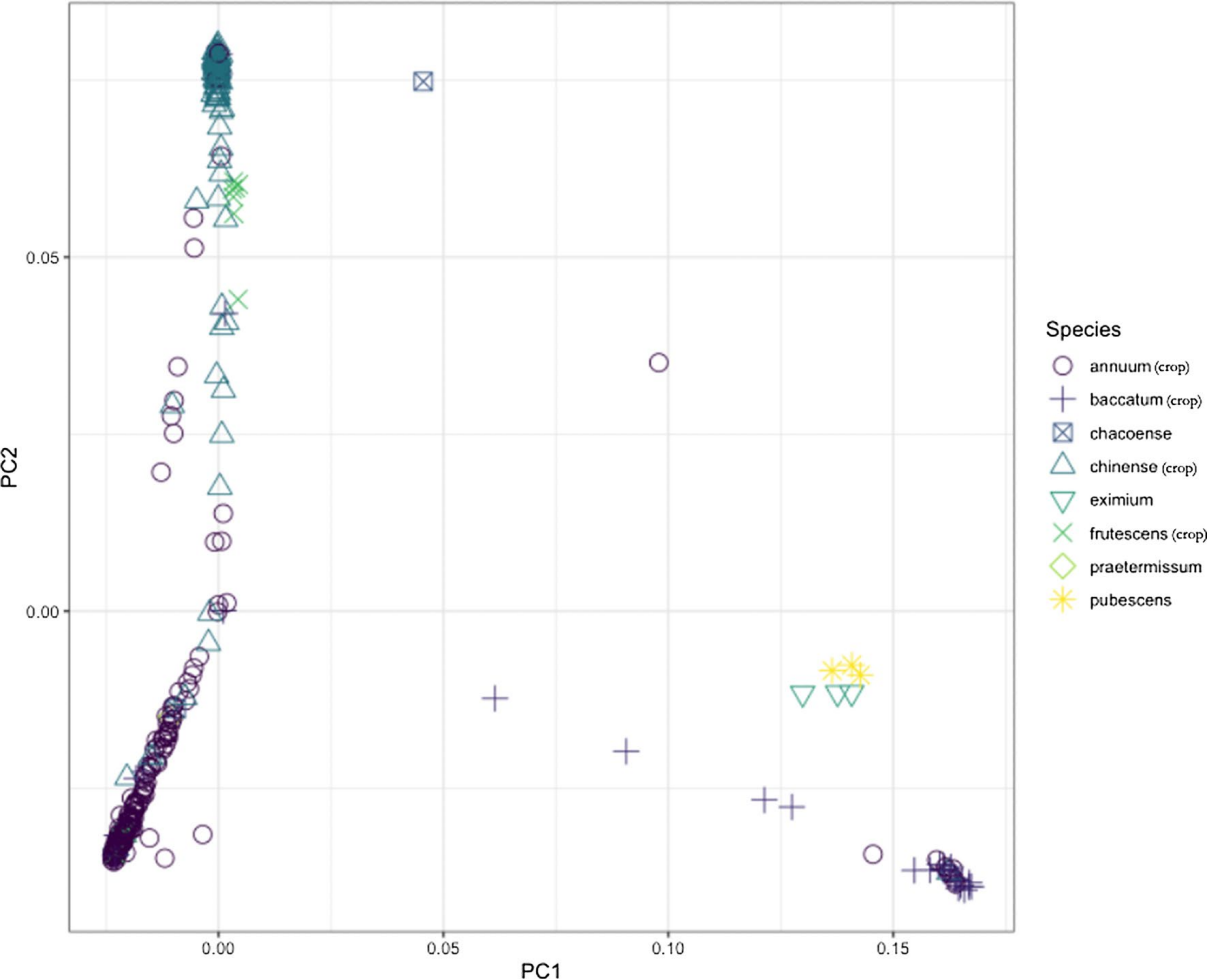
Principal component analysis of 467 accessions representing eight Capsicum species. There are three distinct clusters dominated by C. chinense (top left), C. annuum (bottom left), and C. baccatum (bottom right)

Results from double-digest GBS of 467 pepper accessions yielded 22,916 polymorphic SNPs. These SNPs were distributed throughout the euchromatic and pericentromeric regions, providing even genome-wide coverage (Additional file 1: Fig. S1). PCA projections revealed clear population structure within and between species. The first two principal components explained a combined 17.75% of the genetic variance (Fig. 1; PC1, 9.29% and PC2, 8.46%) and three major groups were identified. The first principal component separated *C. baccatum* accessions (Fig. 1-bottom right) from the other species. The second principal component separated *C. annuum* (Fig. 1-bottom left) from *C. chinense* and *C. frutescens* (Fig. 1-top left). Further, the combined PC1 and PC2 shows potential evidence of interspecific hybridization between the commonly cultivated specie s (*C. annuum*, *C. baccatum*, *C. chinense, C. frutescens*), as seen from individuals located in genetic space between the major cultivated species clusters; however, it is possible that there are other drivers of this pattern. Chiefly, *C. annuum* and* C*. *baccatum* each formed separate groups*,* while *C. chinense* and* C*. *frutescens* formed a distinct group together. Additionally, *C. pubescens* and* C*. *eximium* formed a small group near *C. baccatum* accessions.

### Nucleotide diversity

**Fig. 2:**
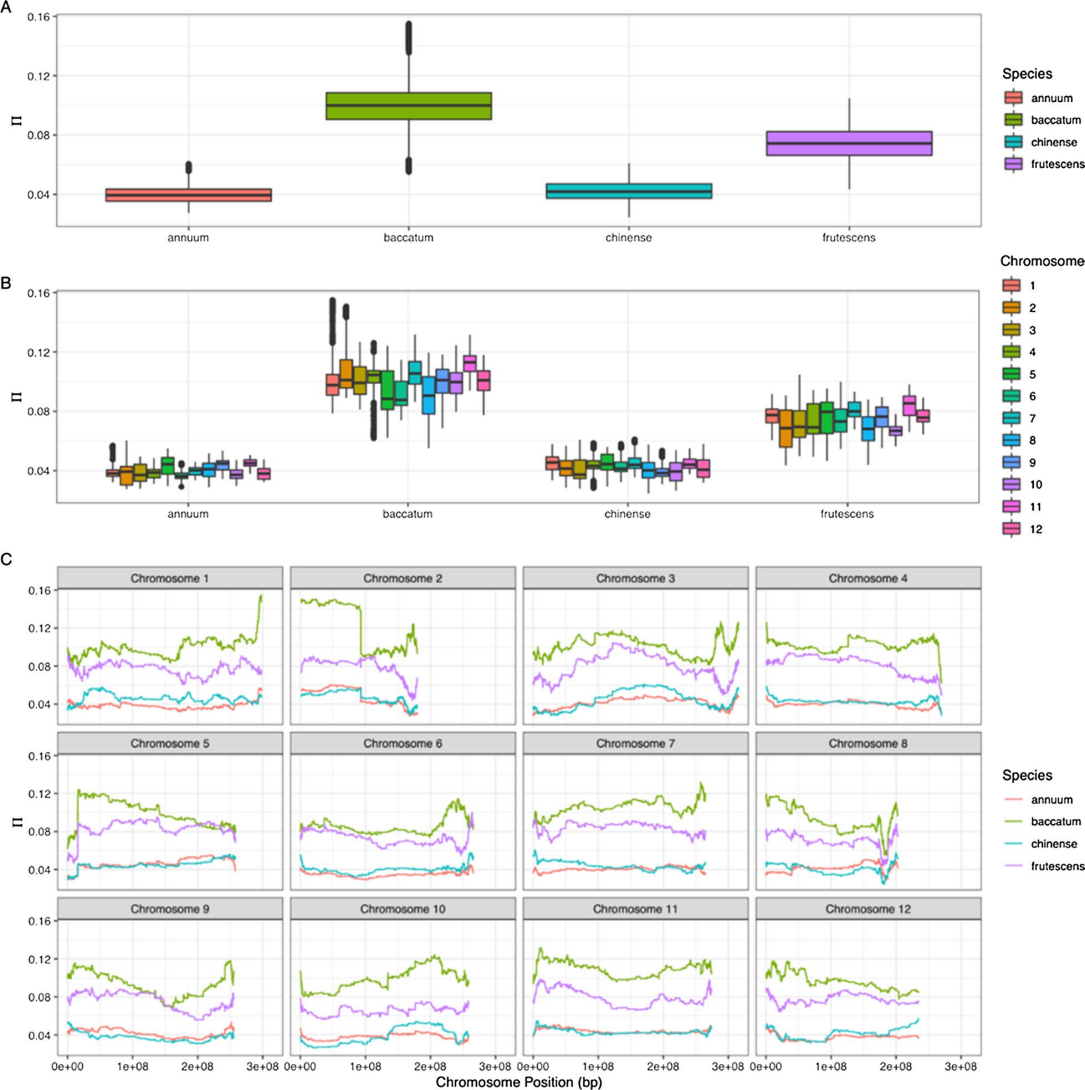
Capsicum nucleotide diversity (π) calculated **A** genome wide by species and **B** chromosome-wide by species. **C** Scans of nucleotide diversity (π) across each chromosome of four species. Box plots show the median, box edges represent the first and third quartiles, and the whiskers extend to farthest data points within the 1.5 × interquartile range outside box edges

There were differences in nucleotide diversity (π) between the four major crop species (*C. annuum, C. chinense, C. baccatum,* and *C. frutescens*, p < 0.001 Dunn test). We estimated π for *C. annuum* to be 0.0404 ± 0.0000387 (se), C. *chinense* to be 0.0432 ± 0.0000470 (se), C. *baccatum* to be 0.102 ± 0.0000977 (se), and *C*. *frutescens* to be 0.0745 ± 0.0000756 (se) (Fig. 2). In addition to genome-wide differences (Fig. 2a), there are large chromosome-wide differences in π values between species for individual chromosomes, as well, especially for chromosome 2 (Fig. 2b). Interestingly, sliding window estimates of π in *C. baccatum* diverge from those of other species most frequently, with π increasing in *C. baccatum* in regions for which π decreases in other species (e.g., Fig. 2c; chromosomes three and six)*.*

### Phylogenetic analysis of plant and fruit phenotypes

**Fig. 3:**
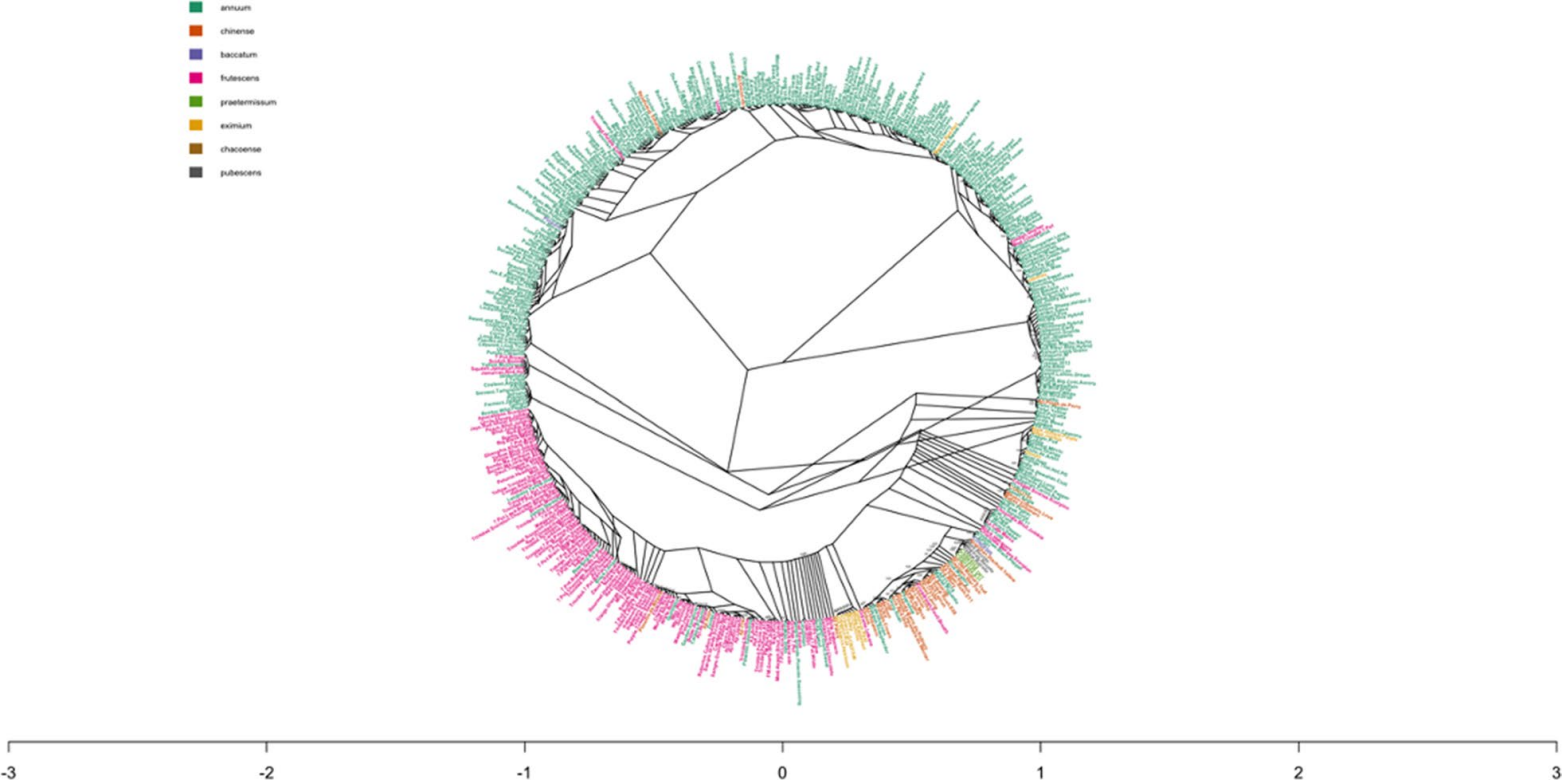
Neighbor joining tree of accessions explored in this manuscript. Colors represent different species assignment based on passport data

This study afforded an opportunity for phylogenetic analysis of 467 pepper accessions sourced from all over the world (Fig. 3). In general, common phenotypes appear to cluster together regardless of where the varieties were developed. For example, accessions with purple color cluster together (Purple Nurple, Fluorescent Purple, Royal Black, Purple Glow in the Dark, and the Black Pearl-Fig. 3). Varieties with variegated leaves clustered together (e.g., Tricolor Variegata, Jigsaw, Rainforest Trifoliage, Calico Hybrid Ornamental, and Var. Black Pearl). The clade containing Shishitou and Hot Shishitou also contain Pepperonia Italian and Pepperoncini Greek accessions, showing relationships between plants with shared phenotypes that were developed in very different geographies. There was also a clade that included many long-fruited varieties (e.g., Big Jim, Monster Chili and Joe E. Parker) indicating that these may also harbor a locus for producing the long fruit phenotype. Further there was a cluster of varieties producing large width and length fruits (e.g., Giant Aconcagua, Cubanelle, Big Sweet Red, and Big Bertha), indicating that while developed in different cultures there may be a similar genetic basis for these commonly selected phenotypes. There are also numerous occurrences of clades that contain clusters of varieties with colored fruits (black, brown, purple, white, yellow, and orange) other than the most common green and red.

Species clustering was consistent with previous population studies in *Capsicum* [24–26]. Nucleotide diversity differed across domesticated species, with evidence of reduced diversity in the more widely cultivated species (*C. annuum* and *C. chinense*). Species groupings in PCA aligned with expectations based on previous research [14, 27–29], with notable admixture suggestions between *C. annuum*, *C. chinense,* and *C. frutescens* (Fig. 1). However, since species were determined based on historic records, species misidentification, as well as actual interspecific hybridization, may contribute to this pattern. Nevertheless, gene flow between species would be consistent with the interspecific hybridization long used in pepper breeding [30]. For example, *C. chinense,* which readily crosses with *C. annuum,* has been used as a source for novel traits [30].

Like previous work, diversity was found to be lower in the more widely cultivated species, *C. annuum* and *C. chinense,* than in *C. baccatum* [13]. The reduction in nucleotide diversity for *C. annuum* and *C. chinense* may be tied to regions under selection during domestication and improvement. The reduction may also be due to limited numbers of parents being the basis of improvement as peppers became common in new geographies across the world [29]. Species genome-wide level differences in nucleotide diversity did not appear to be driven by specific chromosomes; however, there were notable reductions in diversity in *C. annuum* and *C. chinense* on chromosomes with known domestication loci that have previously been identified (e.g., chromosomes two, six, and ten [31]). The different domestic *Capsicum* species have different patterns of diversity but show a potential signal of interspecific hybridization and show some clustering by shared phenotype.

### Limitations

- There is limited information about geographic origin of species
- Limited phenotypic information on samples
- Limited information on origin of cultivar names

## Supplementary Information

**Additional file 1: Figure S1.** Distribution of 22,916 SNPs across 12 chromosomes from genotyping by sequencing of 467 Capsicum accessions. The numbers 0-19 represent the number of SNPs which fall into each 1,000,000 bp bin across each chromosome.

**Additional file 2: Table S1.** Accessions and Species, name from seed provider, and species number used in this study.

## Data Availability

NCBI Sequence Read Archive: https://www.ncbi.nlm.nih.gov/bioproject/PRJNA876725 [20]. Zenodo: https://doi.org/10.5281/zenodo.7268809 [21]
