# Supplementary material for: Population structure in diverse pepper (Capsicum spp.) accessions
Source: BMC Res Notes. 2023 Feb 25;16:20. doi: 10.1186/s13104-023-06293-3 (PMC9960466; doi:10.1186/s13104-023-06293-3)

**Figure S1.** *Distribution of 22,916 SNPs across 12 chromosomes from genotyping by sequencing of 467 Capsicum accessions. The numbers 0-19 represent the number of SNPs which fall into each 1,000,000 bp bin across each chromosome.*


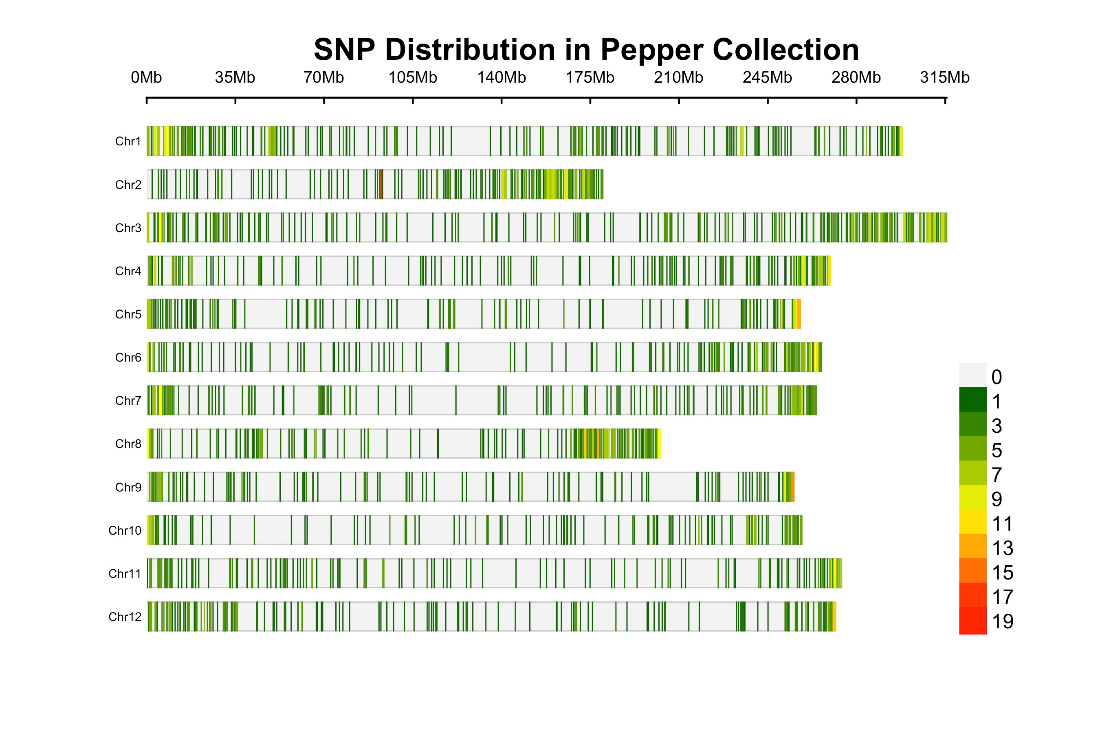

Supplement: Supplementary file 1 — Additional file 1: Figure S1. Distribution of 22,916 SNPs across 12 chromosomes from genotyping by sequencing of 467 Capsicum accessions. The numbers 0-19 represent the number of SNPs which fall into each 1,000,000 bp bin across each chromosome. [file 13104_2023_6293_MOESM1_ESM.docx]
